# Rapid Identification of Patients with Rapidly Progressive Dementia

**DOI:** 10.1002/alz.092437

**Published:** 2025-01-03

**Authors:** Yoav D Piura, Philip W Tipton, S Richard Dunham, Nihal Satyadev, Neill R Graff‐Radford, Gregory S Day

**Affiliations:** ^1^ Mayo Clinic, Jacksonville, FL USA; ^2^ Washington University School of Medicine, Saint Louis, MO USA; ^3^ Department of Neurology, Mayo Clinic, Jacksonville, FL USA

## Abstract

**Background:**

Patients with the precipitous onset of cognitive symptoms (i.e., suspected rapid progressive dementia [RPD]) may continue to decline (true RPD) or may stabilize or improve (non‐RPD). Recognizing patients with RPD early in the disease course is important to support clinical care and enrollment in research.

**Method:**

Patients referred for the evaluation of suspected RPD were prospectively enrolled and followed for up to 2 years at Mayo Clinic in Florida (Jacksonville, FL; Jan‐2020 – Oct‐2023) and Washington University in St. Louis (Saint Louis, MO; Jun‐2016 – Dec‐2019). Clinical data were independently reviewed by two dementia specialists who established clinical diagnoses, referencing published criteria. Patients were diagnosed with RPD if they progressed to dementia (global Clinical Dementia Rating® [CDR] ≥1) within 1 year or incapacitation (CDR ≥2) within 2 years of symptom onset. Univariate statistics compared demographics, clinical details, and test results between patients with RPD and non‐RPD. Variables with p<0.1 were entered into a multivariate logistic regression model to identify factors associated with RPD.

**Result:**

Of 248 patients referred for suspected RPD, 187 (75.4%) met stated criteria for RPD. Patients with RPD were older (62.6±14.4 years vs 55.0±18.3 years; p<0.001), and more likely to be diagnosed with Alzheimer disease or related dementias (28.3% vs 9.8%; p = 0.003) or Creutzfeldt‐Jakob (19.1% vs 4.6%; p = 0.008). Non‐RPD cases were more often attributed to autoimmune/inflammatory disease (42.6% vs 29.4%, p = 0.061) or other causes (e.g., psychiatric, neoplasms, toxic/metabolic; 34.4% vs 16.6%, p = 0.006). After controlling for age, hallucinations (OR, 2.81; 95%CI: 1.21‐6.52), elevated CSF protein (OR, 2.43; 95%CI: 1.17‐5.03), and CSF white blood cell count <5 cells/mm3 (OR, 2.77; 95%CI: 1.32‐5.85) were independently associated with RPD. Cortical visual loss (15/183, 8.2%) and substantial brain atrophy on MRI (27/184, 14.7%) were specific to patients with RPD, representing additional distinguishing features.

**Conclusion:**

Hallucinations, cortical visual loss, substantial atrophy on MRI, elevated CSF protein, and normal CSF white blood cell count were independently associated with RPD. Early detection of these features in patients with suspected RPD may inform clinical care and enrollment in research studies of RPD.

